# Genome-Wide Approach of Gene–Nutrient Intake Interaction Study for Essential Hypertension in a Large Korean Cohort (KoGES)

**DOI:** 10.3390/nu16234147

**Published:** 2024-11-29

**Authors:** Youhyun Song, Ja-Eun Choi, Jae-Min Park, Yu-Jin Kwon, Kyung-Won Hong, Ji-Won Lee

**Affiliations:** 1 Department of Family Medicine, Gangnam Severance Hospital, Yonsei University College of Medicine, Seoul 06273, Republic of Korea; wlgmeo_o@yuhs.ac; 2 Healthcare Research Team, Health Promotion Center, Gangnam Severance Hospital, Yonsei University College of Medicine, Seoul 06273, Republic of Korea; 3 Advanced Institute of Technology, Theragen Health Co., Ltd., Seongnam-si 13493, Republic of Korea; jaeun.choi@theragenhealth.com; 4 Department of Family Medicine, Uijeongbu Eulji Medical Center, Eulji University, Uijeongbu 11759, Republic of Korea; milkcandy@naver.com; 5 Department of Family Medicine, Yongin Severance Hospital, Yonsei University College of Medicine, Yongin-si 16995, Republic of Korea; digda3@yuhs.ac; 6 Department of Family Medicine, Severance Hospital, Yonsei University College of Medicine, Seoul 03722, Republic of Korea; 7 Institute for Innovation in Digital Healthcare, Yonsei University, Seoul 06237, Republic of Korea

**Keywords:** blood pressure, hypertension, gene-nutrient interaction, genome-wide association study

## Abstract

**Background/Objectives:** There is limited evidence on gene-nutrient interaction associated with hypertension (HTN). We examined interactions between genotypes and various nutrients that influenced high blood pressure (BP). **Methods:** Data were obtained from a total of 50,808 participants from the Korean Genome and Epidemiology Study (KoGES). Dietary intake was assessed by a food frequency questionnaire, and dietary reference intakes (DRIs) were set. We performed genome-wide association analyses (GWAS) and subsequent interaction analyses with genome-wide significant SNPs to identify genomic loci that interact with specific nutrients associated with HTN. **Results:** We identified one locus near the CUB and Sushi Multiple Domains 1 (CSMD1) gene that showed interaction with dietary iron and vitamin B6 (Vit.B6) intake and significantly influenced HTN risk. Among the individuals consuming iron above DRI (9.5 mg/day for men, 9.25 mg/day for women), carriers of the rs13282715 minor allele (A) at 8p23.2 showed a lower risk of HTN than those who did not (odds ratio [OR] 0.723, 95% confidence interval [CI] (0.644–0.813), *p*-value 4.858 × 10^−8^; interaction *p*-value 1.336 × 10^−3^). Among the individuals consuming Vit.B6 above DRI (1.5 mg/day for men, 1.4 mg/day for women), carriers of the same variant rs13282715 minor allele (A) also showed a lower risk of HTN (OR 0.733, 95% CI 0.733 (0.656–0.819), *p*-value 4.121 × 10^−8^; interaction *p*-value 7.183 × 10^−4^). **Conclusions:** We identified a novel gene-nutrient interaction regarding dietary iron and Vit.B6 intake affecting the risk of HTN in Korean adults. This suggests individuals with the variant may benefit from lower HTN risk from dietary intervention of iron and Vit.B6 intake. Further studies with larger diverse populations are warranted to validate our findings.

## 1. Introduction

Hypertension (HTN) or high blood pressure (BP) affects approximately 32% of adults worldwide [1,2]. It is an established important risk factor for cardiovascular disease (CAD), cerebrovascular disease (CVD), and chronic kidney disease [3]. Accumulating evidence suggests its association with non-vascular diseases such as cancer, dementia, and osteoporosis, among numerous others [4]. Korea, in particular, due to the rapidly aging population and increased life expectancy, has seen the total number of HTN individuals rising as well as its related complications. The population prescribed antihypertensive medication has increased 4.1-fold between 2002 and 2021, with the most common regimen being dual therapy using ACEi/ARB and CCBs [5]. 

Risk factors for HTN consist of genetic and lifestyle factors. Although twin studies have shown that heritability may account for up to 34–67% of BP estimations [6], the collective effect of all BP loci identified through genome-wide association studies (GWAS) until recently could explain only up to 20% of BP variability at most [7,8]. Previous studies have suggested that the failure to account for gene-gene and gene-environment interactions is one of the possible causes that lead to the underestimation of heritability in GWAS [9].

Relatedly, lifestyle modification is fundamental for both the prevention and treatment of HTN, and major guidelines have outlined the significance of non-pharmacological therapy, especially dietary interventions [3,10]. Various dietary modifications such as salt restriction, “healthy diets” such as the DASH diet, and weight reduction have all been proven to be effective in lowering BP; however, most have shown considerable interpersonal variations in changes in BP [11,12]. Such variations may also be partly related to gene-gene [3,7,12] or gene-environment and gene-nutrient interactions [13,14,15].

Although the identification of gene-nutrient interactions could provide novel insights into the development of HTN, there are few studies, and they mainly regard “major” dietary factors. To the best of our knowledge, this is the first study performed to evaluate a wide range of nutrients, including micronutrients or “non-major” dietary factors, gene variability, and gene-nutrient interactions in HTN. Additionally, there has been a paucity of such research in Asian populations. Therefore, we aimed to identify novel genetic variants influencing HTN with significant gene-nutrient interaction, using a large-scale GWAS of Korean adults.

## 2. Methods

### 2.1. Study Population and Design

**Figure 1:**
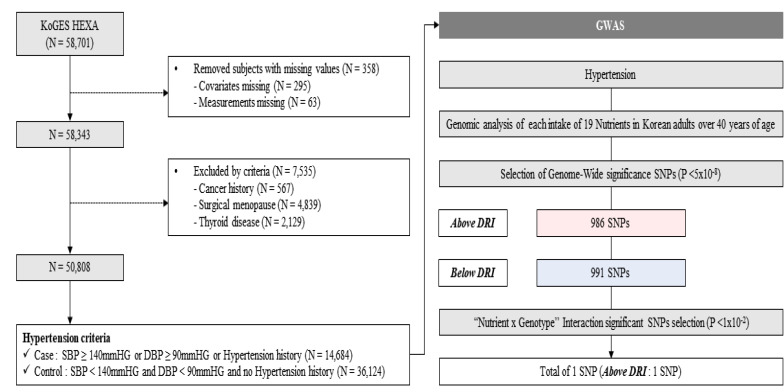
Study design. KoGES HEXA, Korean Genome and Epidemiology Study-Health Examinee Study; N, number; SBP, systolic blood pressure; DBP, diastolic blood pressure; GWAS, genome-wide association study; SNP, single nucleotide polymorphism; P, *p*-value; DRI, Dietary Reference Intake.

Participants were part of KoGES, a government-funded study platform including large population-based cohorts designed to identify genetic and environmental factors of common chronic diseases in Korean adults (National Research Institute of Health, Ministry of Health and Welfare, and Centers for Disease Control and Prevention). The KoGES recruited community dwellers and subjects from the national health examinee registry who were over 40 years of age at baseline. Further information regarding KoGES has been previously described in detail elsewhere [16]. All participants signed an informed consent form before the study, and the present study was performed in accordance with the Declaration of Helsinki and approved by the Institutional Review Board of Theragen Bio (Approval Numbers: 700062-20190819-GP-006-02). In total, the data of 58,701 subjects, for whom genome-wide single nucleotide polymorphism (SNP) genotype data were available, were included in our initial dataset. Figure 1 depicts the overall study design.

### 2.2. Genotyping and Covariates

Genomic DNA was extracted from peripheral blood samples, and the extracted DNA was genotyped using Korea Biobank arrays (KoreanChip). Details regarding KoreanChip have been previously described [17]. To control the quality of the genotyping results, SNPS were excluded before imputation if they did not meet the following criteria: call rate > 97%, missing genotype < 0.01, Hardy-Weinberg equilibrium *p* > 0.000001, and minor allele frequency > 0.01.

Participants’ anthropometric measurements were performed by trained technicians, and lifestyle data were collected by trained interviewers by questionnaires. Body mass index (BMI) was computed as weight (kg)/height (m) squared, and waist circumference (WC) measurements were taken midway between the lowest rib and the top of the iliac crest. Blood samples were obtained after a minimum of 8 h of fasting. Alcohol consumption (g/day) was calculated based on the frequency of types of alcoholic beverages consumed to consider alcoholic content and how many drinks were consumed per occasion. Smoking status was categorized into non-smoker, past smoker, and current smoker. Regular exercise status was defined as the routine performance of 30 min or more of exercise per day. Blood pressure was measured by trained technicians using standard mercury sphygmomanometers (Baumanometer-Standby; W.A. Baum Co. Inc., New York, NY, USA). Systolic blood pressure (SBP), diastolic blood pressure (DBP), and pulse rate (PR) were measured in a sitting position after a minimum of 5 min of rest at least twice, and values were defined as the average of the left and right arms [18]. Hypertension (HTN) was defined as SBP ≥ 140 mmHg or DBP ≥ 90 mmHg, or diagnosis of HTN [19]. Diabetes mellitus (DM) was defined as fasting serum glucose ≥ 126 mg/dL or HbA1c ≥ 6.5%, or a DM diagnosis. Dyslipidemia was defined as total cholesterol (TC) ≥ 200 mg/dL, or triglycerides (TG) ≥ 150 mg/dL, or low-density lipoprotein cholesterol (LDL-C) ≥ 130 mg/dL, or high-density lipoprotein cholesterol (HDL-C)  <  40 mg/dL (for men) and HDL-C  <  50 mg/dL (for women), or a prior diagnosis.

### 2.3. Determination of Nutrition Intake Reference

A semi-quantitative food frequency questionnaire (FFQ) involving 103 items was developed for the KoGES to assess the usual dietary intakes of Korean adults who participated [16,20]. Consumption frequency and portion size of foods consumed over the past year were reported by the participants via the FFQ. Our nutrition intake criteria were set mainly based on the 2020 Korean Dietary Reference Intakes (DRIs) [21]. DRI is a general term for a collection of reference values utilized in planning and assessing nutrient intakes of healthy individuals. For macronutrients (i.e., carbohydrates, protein, and fat), we defined the upper limit of each macronutrient’s acceptable macronutrient distribution range (ADMR) as the intake reference criteria. The DRI of cholesterol was set based on the reference value derived from chronic disease endpoints [22]. The DRIs of sodium (Na), potassium (K), vitamin E, and fiber were set based on adequate intake (AI), the recommended average daily nutrient intake level derived from approximations of observed mean intake by healthy people or experimentally derived intake levels [23]. The DRIs of other micronutrients were set based on recommended nutrient intakes (RNIs), the average level of daily nutrient intake sufficient to meet the requirements of most (97–98%) healthy people. DRIs vary by sex and age, and since KoGES included participants over 40 years of age, we calculated the mean values of the DRIs of the over-40-year-old population for each gender. Specific criteria of the DRIs used in this study are shown in Supplementary Table S1.

### 2.4. Statistical Analysis

To compare the differences between the HTN case group and the control group, we used an independent two-sample t-test for continuous variables and a chi-square test for categorical variables. Data are presented as mean ± standard deviation (SD) or number (percentage of the total sample). Principal component (PC) analysis was performed to reduce genomic bias referring to the region where samples were collected, and PC1 and PC2 were used as covariates in statistical analyses.

For each of the 19 nutrient groups (for both above DRI and below DRI), we conducted three independent GWASs to identify variants associated with HTN: (1) all subjects, (2) above DRI group, and (3) below DRI group. At this stage, we analyzed GWAS results for both the HTN group and the control group. All GWASs were conducted using logistic regression analysis and were adjusted for age, sex, alcohol consumption, smoking status, exercise status, total energy consumption, PC1, and PC2 implemented in PLINK version 1.9. SNPs with genome-wide significance (*p*-value < 5 × 10^−8^) within each nutrient intake group (above or below) were selected among the GWAS results. Then, among those SNPs, we further selected those that were uniquely significant in a single nutrient group. With these selected SNPs, interaction analysis for HTN was conducted using the generalized linear model of R statistics (Version 4.0.3; R Foundation for Statistical Computing, Vienna, Austria). Finally, the SNP with significant gene-nutrient interaction (*p*-value < 5 × 10^−2^) associated with HTN was identified from our analysis.

## 3. Results

This study utilized data from 58,701 Korean adults over 40 years old from the KoGES cohort. Notably, 358 participants with missing covariates or measurements and 7535 participants with histories of cancer, surgical menopause, and thyroid diseases were excluded. 50,808 subjects were included for the GWAS and subsequent analyses, categorized into HTN cases (*n* = 14,684) and controls (*n* = 36,124) as shown in Figure 1. The target disease was HTN, and the target nutrients were the 19 major nutrients investigated in the KoGES. After the initial GWASs, a total of 1011 SNPs (986 SNPs above DRI and 991 SNPs below DRI) showed genome-wide significance. Within these significant SNPs, we identified 1 SNP related to HTN above DRI of iron and Vit.B6, with significant nutrition-by-gene interaction.

### 3.1. Population Characteristics

Table 1 shows the general characteristics of the study population according to hypertension status. The mean age of the total population was 53.59 ± 8.12 years, and 61.4% were female. Of the 50,808 participants, 14,684 (28.9%) had HTN, and 36,124 (71.1%) did not have HTN. Subjects with HTN were older, comprised a larger proportion of males and current/ex-smokers, and consumed more alcohol, but a smaller proportion of participants engaged in regular exercise than those without. In addition, the HTN group showed, by definition, higher SBP and DBP as well as a higher pulse rate, BMI, WC, glucose, HbA1c, and TG. Serum HDL-C and TC were lower in participants with HTN. Incidences of DM, DL, CAD, and CVD were all higher in the HTN group. All values, with the exception of dietary sodium, showed significant differences between the HTN and control groups (*p* < 0.01).

### 3.2. Nutritional Intake

As shown in Table 1, the control group without HTN consumed more total calories than the HTN group, although the mean difference was minimal at approximately 39 kcal/day. Accordingly, the intake of micronutrients per day was also minimally higher in the subjects without HTN. However, those with HTN showed a higher percentage of carbohydrate intake and a lower percentage of protein intake compared to those without. For a detailed nutrition intake analysis, refer to Supplementary Table S1, which depicts the proportions of subjects with nutrition intake above or below the reference criteria for each of the 19 major nutrients analyzed in this study. The nutrient intake criteria were set in a sex-specific manner. More than 80% of both genders consumed carbohydrates above the ADMR, and 98~99% consumed protein and fat below the DRI. Most micronutrients, with the exception of P and sodium, were consumed at levels lower than the RNI or AI by the majority of both sexes. The proportion of subjects consuming iron and vitamin B6 above and below the RNIs was similar in both females and males.

### 3.3. HTN, Iron and Vit.B6 Intake, and Genotype

**Figure 2:**
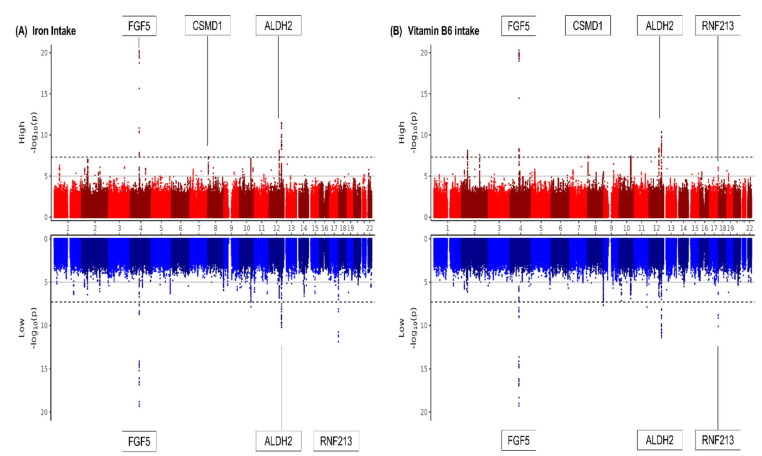
Miami plots showing *p*-values for the SNP associations with hypertension in subjects according to dietary intake of significant nutrients. (**A**) Iron and HTN. (**B**) Vitamin B6 and HTN. Genome-wide significant *p*-value criteria (*p*-value < 5 × 10^−8^) are shown by dotted lines, and genome-wide suggestive *p*-value criteria (5 × 10^−8^ ≤ *p*-value < 1 × 10^−5^) are shown in solid lines. The y-axis is –log_10_(*p*-value) of the single nucleotide polymorphisms (SNPs) and the x-axis is chromosomal position (hg19). Candidate genes were identified as the genes nearest to the lead SNPs with nearby variant clusters showing similar significance.

**Figure 3:**
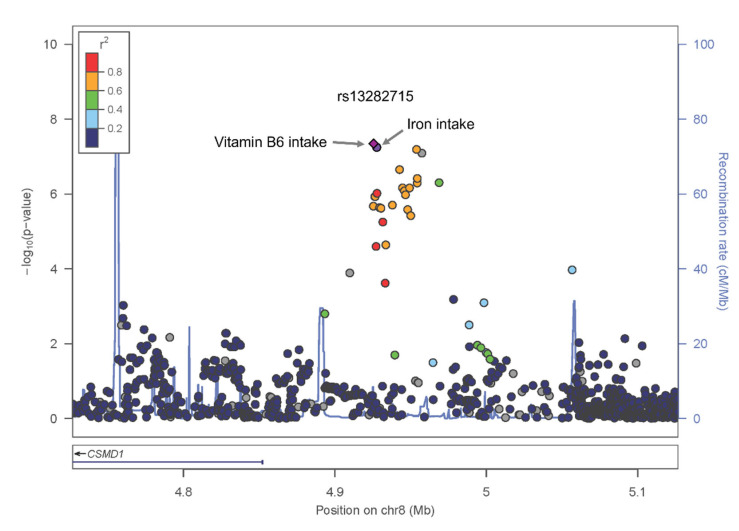
Signal plot of the SNP interacting with iron and Vit.B6 associated with HTN. SNP is plotted by chromosomal position (hg19; x-axis) and association with HTN from the current study (–log_10_(*p*-value); y-axis).

One variant was identified to be uniquely significant (i.e., not showing widespread significance over nutrients and showing unilateral significance in either above or below DRI) in two nutrient groups, iron and vitamin B6. Results of the two significant GWASs are depicted in Figure 2 as Miami plots using log10 transformed *p*-values. The GWAS of HTN-iron (Figure 2A) showed one genome-wide significant variant, rs13282715 (8p23.2). The GWAS of HTN-vitamin B6 (Figure 2B) showed the same variant, rs13282715 (8p23.2), to show genome-wide significance. The regional association plot for the novel SNP rs13282715 is shown in Figure 3, showing its location near CSMD1. GWAS and gene-nutrient interaction analyses are shown in their entirety in Supplementary Tables S2 and S3.

### 3.4. Nutrient-by-Gene Interaction Analysis Associated with HTN

Table 2 shows the SNP with significant nutrient-by-gene interaction associated with HTN. A single SNP was identified to be associated independently with two nutrients and HTN: iron and Vit.B6. Among the individuals consuming iron above DRI (9.5 mg/day for men, 9.25 mg/day for women), carriers of CUB and Sushi Multiple Domains 1 (CSMD1)- rs13282715 minor allele (A) showed a lower risk of HTN than those who did not (odds ratio [OR] 0.723, 95% CI (0.644–0.813), *p*-value 4.858 × 10^−8^; interaction *p*-value 1.336 × 10^−3^). Among the individuals consuming Vit.B6 above DRI (1.5 mg/day for men, 1.4 mg/day for women), carriers of the same CSMD1- rs13282715 minor allele (A) also showed a lower risk of HTN than those who did not (OR 0.733, 95% CI 0.733(0.656–0.819), *p*-value 4.121 × 10^−8^; interaction *p*-value 7.183 × 10^−4^). When analyzed for association with HTN independently, the SNP did not show genome-wide significance. In Model 2, Type 2 diabetes mellitus and dyslipidemia were included as additional covariates in the calculations for *p*-values and interaction *p*-values, based on the original Model 1. Supplementary Table S4 shows additional gender-specific analyses as Model 3. All models showed similar trends but did not hold statistical significance, which may have been caused by smaller samples. When compared by gender, females showed higher values of association than men.

## 4. Discussion

In this genome-wide interaction analysis of the KOGES cohort, we identified a novel variant, rs13282715 near CSMD1 (8p23.2), that modified the association between dietary iron and Vit.B6 intake with HTN. This study showed that Korean adults carrying the risk allele A at rs13282715 had a lower risk of HTN when consuming either iron or Vit.B6 above the DRIs. Although limitations exist in our study, to the best of our knowledge, this is the first study to identify gene-diet interactions in HTN regarding essential micronutrients besides sodium.

Various studies have suggested genes that interact with specific environmental factors to affect HTN risk or BP. As reviewed by Kokubo et al. [9] and the authors of this study, regarding dietary factors associated with gene interactions, the majority of studies were performed regarding salt intake [15,24,25,26,27,28,29]. A few more studies were conducted regarding “healthy diets” such as the DASH diet score [30,31], dietary fat [32] or fatty acid intake [33,34], and dietary protein [35] or high energy intake [36]. Micronutrients such as iron or Vit.B6 identified in this study can be easily adjusted if needed; thus, further studies with larger populations and non-Southeast Asian populations are fully warranted.

The CSMD1 gene, named for its repeated CUB and Sushi domains, encodes a transmembrane protein of the vacuolar-protein-sorting-13 family. Although the function of this protein has not been clarified in humans, CUB and Sushi domains are found in various proteins and are speculated to be sites of protein-protein or protein-ligand interactions [37,38]. Until now, the exact pathophysiology between CSMD1 and BP regulation has not yet been clarified. However, CSMD1 has previously been identified to be associated with HTN risk in Koreans [39] and Africans [40], as well as to affect SBP response to hydrochlorothiazide [41] and candesartan [42]. Other studies have found the gene to be related to metabolic syndrome [43] and peripheral artery disease [44].

Iron [45] and Vit.B6 [46] are both essential micronutrients for a wide range of metabolic and regulatory processes of the human body, such as erythropoiesis, inflammatory response, muscle and lipid metabolism, among many others. Decreased dietary intake of both has been identified to be associated with BP or HTN, respectively, in previous studies. Regarding iron, the results have been inconsistent; the International Collaborative Study on Macro-/micronutrients and Blood Pressure study [47] and another study by Galan et al. [48] showed inverse associations between dietary intake of total iron and BP or HTN risk, while other studies have found U-shaped associations [49,50] or a null association [51]. The mechanism underlying this association has not been clarified in detail, though low dietary iron intake may contribute to iron deficiency (ID), which produces reactive oxygen species (ROS) and activates chronic inflammation [52], as well as affects endothelial dysfunction [53] by elevated expression of the vasoconstrictor endothelin-1 [54]. Iron deficiency (ID) is relatively common, and the detrimental effect of ID on prognosis in a broad range of CADs and CVDs has been receiving increasing attention [55,56].

Our finding is in agreement with those from previous studies regarding Vit.B6. Of the many functions of Vit.B6, one of the most widely discussed is its role in regulating BP, atherosclerosis, and CVD/CAD [57,58]. Low serum Vit.B6 levels have been shown to be associated with HTN in numerous studies [59,60,61,62]. Randomized controlled trials have additionally shown high-dose Vit.B6 supplementation to significantly lower BP [63,64]. The active form of Vit.B6, pyridoxal phosphate (PLP) acts as a cofactor for around 160 reactions in the human body. Vit.B6 deficiency results in alterations in the synthesis of neurotransmitters such as serotonin and dopamine, which leads to a reduction of phospholipids in the brain and thus affects Ca2+ transport and metabolism, causing positive regulation of BP. Also, a deficit in Vit.B6 may lead to elevated levels of homocysteine, which is correlated with endothelial dysfunction, CVD burden, and HTN [65]. In addition, Vit.B6 has also been associated with enhanced diuretic activity and enhanced glutathione production, which may affect BP [46,66].

While iron and Vit.B6 have not been identified to be in direct interaction, they indirectly influence each other’s metabolism and function and share common physiologic pathways regarding HTN. ID can impair the absorption and utilization of Vit.B6, and Vit.B6 deficiency in turn may impair heme synthesis. They both play major roles in the heme synthesis pathway, increased oxidative stress and inflammation, nitric oxide production, endothelial dysfunction, and regulation of the renin-angiotensin-aldosterone system, which may be how they jointly contribute to high BP.

There are a few limitations in this study. First, the cross-sectional design cannot discern reverse causation in which subjects with HTN may have changed their dietary patterns to better control their BP. Second, FFQ was utilized to assess nutritional intake. Although FFQ is a widely used tool to assess nutritional status in large populations, there are inaccuracies regarding absolute nutrient values, a lack of detail in certain foods, and the self-reported nature of the tool itself, which is subject to bias. In addition, this study used a 103-item FFQ developed for the KOGES, which was appropriate given our study population; however, future studies with diverse ethnicities using a common FFQ are warranted for generalization and replication. Also, the variant we identified showed MAFs of 0.2018 in Europe and 0.117 in America, rendering it relatively “common”, which gives it more validation for further evaluation in Western populations.

Despite the limitations, this study has several unique strengths. First, this study is the first to identify specific gene-nutrient interactions associated with HTN regarding iron and Vit.B6. Previous studies have identified “major” gene-nutrient interactions, but micronutrients, with the exception of salt, have not been explored, to the best of our knowledge. Micronutrient deficiencies are highly prevalent, readily quantifiable in clinical practice, and can be easily modified by dietary intervention or supplementation. Additionally, other studies have identified gene-nutrient interactions but could not or did not specify whether the gene variant influenced a specific nutrient or whether its effect was nutritionally widespread. In this study, we analyzed the effects of various nutrients simultaneously, by which we were able to select variants with nutrient specificity. Moreover, the findings of this study provide novel insights into gene-nutrient interactions of HTN utilizing GWASs with a large real-world database representative of the Korean adult population. A more comprehensive understanding of gene-nutrient interactions for specific diseases will potentially pave the way to “precision nutrition” based on tailored dietary recommendations according to individual genotypes. With further studies and a more easily accessible gene testing environment in the future, carriers of the risk allele A in rs13282715 may be subject to nutritional counseling for higher iron and Vit.B6 intake throughout their lifetime, preferably starting from the individuals’ early stages of life.

## 5. Conclusions

We demonstrated novel gene-nutrient interactions for HTN. Dietary iron and vitamin B6 intake above the DRI significantly interacted at the CSMD1 locus (rs13282715, 8p23.2) in relation to HTN with genome-wide significance (ORs: 0.72 for iron and 0.73 for vitamin B6). These results suggest that individuals with the variant may benefit from a lower HTN risk from dietary intervention of increased iron and vitamin B6 intake. Larger studies with more diverse populations are needed to validate and generalize our findings.

## Figures and Tables

**Table 1:** General characteristics of the study population.

	Total	HTN	non-HTN
N	50,808	14,684	36,124
Age, years	53.59 ± 8.12	57.27 ± 7.57	52.09 ± 7.86
Sex (Female), *n* (%)	31,213(61.43)	7494(51.04)	23,719(65.66)
**Lifestyle**			
Alcohol intake, g/day	57.21 ± 176.44	74.98 ± 202.81	49.99 ± 163.97
Smoking status: Never/Quit/Current (*n*, %)	35,796(70.45)/8806(17.33)/6170(12.14)	9368(63.84)/3507(23.90/1799(12.26)	26,428(73.21)/5299(14.68)/4371(12.11)
Exercise status: No/Yes (*n*, %)	23,136(45.54)/27,672(54.38)	6349(43.24)/8335(56.76)	16,787(17.46)/19,337(82.54)
**Disease Prevalence**			
Type 2 Diabetes: Yes/No (*n*, %)	45,887(90.31)/4921(9.69)	12,114(82.5)/2570(17.5)	33,773(93.49)/2351(6.51)
Dyslipidemia: Yes/No (*n*, %)	27,268(53.69)/23,518(46.31)	6570(44.78)/8103(55.22)	20,698(57.31)/15,415(42.69)
Cardiovascular disease: Yes/No (*n*, %)	49,322(97.14)/1452(2.86)	13,945(95.06)/724(4.94)	35,377(97.98)/728(2.02)
Cerebrovascular disease: Yes/No (*n*, %)	50,160(98.78)/622(1.22)	14,292(97.4)/382(2.60)	35,868(99.34)/240(0.66)
**Anthropometric traits**			
BMI, kg/m^2^	23.90 ± 2.87	25.03 ± 2.93	23.44 ± 2.71
Waist Circumference, cm	80.91 ± 9.03	84.52 ± 8.87	79.45 ± 8.68
SBP, mmHg	122.47 ± 15.02	135.2 ± 14.71	117.3 ± 11.70
DBP, mmHg	75.82 ± 9.92	83.33 ± 10.0	72.76 ± 8.10
Pulse, count/min	69.21 ± 9.17	70.04 ± 9.85	68.88 ± 8.86
**Biochemical traits**			
Serum glucose, mg/dL	95.27 ± 19.87	100.56 ± 23.6	93.12 ± 17.69
HbA1c, %	5.71 ± 0.72	5.91 ± 0.88	5.63 ± 0.63
Total cholesterol, mg/dL	197.0 ± 35.62	195.99 ± 36.78	197.41 ± 35.12
Triglycerides, mg/dL	125.96 ± 86.65	145.07 ± 97.3	118.19 ± 80.64
HDL-C, mg/dL	53.53 ± 13.14	51.37 ± 12.65	54.41 ± 13.24
Hemoglobin, g/dL	13.94 ± 1.49	14.26 ± 1.44	13.81 ± 1.50
MCV, fL	91.39 ± 4.74	91.61 ± 4.32	91.3 ± 4.90
MCH, pg	30.47 ± 1.98	30.68 ± 1.75	30.39 ± 2.07
Blood Urea Nitrogen, mg/dL	14.48 ± 3.96	15.21 ± 4.37	14.19 ± 3.74
Creatinine, mg/dL	0.82 ± 0.22	0.86 ± 0.27	0.8 ± 0.19
**Nutrient**			
Total energy, kcal/day	1692.05(1415.28–2017.06)	1669.38(1408.44–1988.18)	1700.20(1418.53–2029.29)
CHO (%)	72.44(67.64–76.52)	73.18(68.41–77.05)	72.13(67.37–76.27)
Protein (%)	13.044(11.63–14.76)	12.91(11.52–14.67)	13.10(11.68–14.80)
Fat (%)	13.32(10.12–17.10)	12.61(9.59–16.28)	13.63(10.35–17.39)
Ca, mg/day	394.03(272.55–551.20)	381.35(264.15–536.90)	399.00(276.30–555.94)
P, mg/day	835.77(664.46–1046.30)	821.23(652.52–1025.18)	841.40(668.93–1052.81)
Iron, mg/day	9.06(6.96–11.83)	8.86(6.80–11.62)	9.14(7.03–11.92)
K, mg/day	2058.35(1553.73–2693.69)	2010.56(1513.78–2649.49)	2079.79(1569.70–2710.63)
Vitamin A (R.E)	397.26(271.16–580.82)	389.89(262.86–574.95)	400.40(274.65–582.99)
Na, mg/day	2246.36(1498.13–3082.89)	2253.01(1487.17–3086.66)	2243.31(1502.91–3080.85)
Vitamin B1, mg/day	0.93(0.73–1.19)	0.90(0.71–1.16)	0.93(0.74–1.20)
Vitamin B2, mg/day	0.83(0.61–1.09)	0.80(0.59–1.06)	0.84(0.62–1.10)
Niacin, mg/day	13.48(10.65–17.11)	13.19(10.39–16.71)	13.60(10.75–17.27)
Vitamin C, mg/day	91.52(61.04–133.48)	88.15(58.64–128.55)	92.89(61.97–135.30)
Zinc, mg/day	7.35(5.89–9.26)	7.26(5.80–9.14)	7.38(5.92–9.31)
Vitamin B6, mg/day	1.46(1.15–1.86)	1.44(1.13–1.84)	1.47(1.16–1.87)
Folate, mcg/day	192.63(140.68–259.45)	189.43(137.66–255.77)	193.85(141.96–261.13)
Fiber, g/day	5.22(3.86–6.87)	5.19(3.81–6.78)	5.24(3.88–6.90)
Vitamin E, mg/day	7.23(5.30–9.81)	7.01(5.13–9.49)	7.33(5.38–9.95)
Cholesterol, mg/day	141.72(88.83–219.39)	132.39(81.42–207.22)	145.89(92.20–224.50)

HTN, hypertension; SBP, systolic blood pressure; DBP, diastolic blood pressure; BMI, body mass index; HbA1c, hemoglobin A1c; HDL-C, high-density lipoprotein cholesterol; MCV, mean corpuscular volume; MCH, mean corpuscular hemoglobin; CHO, carbohydrate; Ca, calcium; K, potassium; P, phosphorus; R.E, retinol equivalents. Data were represented as mean ± standard deviations or median (interquartile range) for continuous variables and number (%) for categorical variables.

**Table 2:** Single nucleotide polymorphism (SNP) showing significant interactions with nutrients associated with hypertension.

SNP	Chr: bp	Alleles		Positional Candidate Gene	Minor Allele Frequency				Coding Allele	Coding Allele Frequency	Gene and Nutrient Association Results				Gene Association Result to Hypertension	
		REF	ALT		Korean	EAS	EUR	AMR			Above Iron		Above Vit.B6		OR (95% CI)	*p*-Value
											OR(95% CI) (*p*-Value)	Interaction *p*-Value	OR(95% CI) (*p*-Value)	Interaction *p*-Value		
rs13282715	8:4926756	T	A	CSMD1	0.065	0.0615	0.2018	0.117	A	0.040	Model 1					
											0.72 (0.64–0.81) **(4.86 × 10^−8^)**	1.34 × 10^−3^	0.73 (0.66–0.82) **(4.12 × 10^−8^)**	7.18 × 10^−4^	0.88 (0.82–0.95)	9.21 × 10^−4^
											Model 2					
											0.74 (0.64–0.87) (1.49 × 10^−4^)	1.26 × 10^−1^	0.74 (0.64–0.86) (6.22 × 10^−5^)	1.38 × 10^−1^	0.89 (0.81–0.98)	1.83 × 10^−2^

SNP, single nucleotide polymorphism; Chr, chromosome; BP, base pair; EAS, East Asian; EUR, European; AMR, American; OR, odds ratio; CI, confidence interval. *p*-values were calculated using logistic regression analysis adjusting for age, sex, exercise, smoking, alcohol intake (g/day), total calorie consumption, PC1, and PC2. Interaction *p*-values were calculated through the interaction term of the general linear regression model. In Model 2, Type 2 diabetes mellitus and dyslipidemia were included as additional covariates in the calculations for *p*-values and interaction *p*-values, based on Model 1.

## Data Availability

Data and materials derived from the Korean Genome and Epidemiology Study (KoGES) have been deposited at the one-stop portal services of the Korea Biobank Network (KBN), available at “https://biobank.nih.go.kr/eng/cmm/main/mainPage.do (accessed on 26 November 2024)”. Researchers may obtain genomic data after approval of relevant authorities as guided in the portal.

## Supplementary Materials

The following supporting information can be downloaded at: https://www.mdpi.com/article/10.3390/nu16234147/s1, Table S1: Reference nutrient criteria and distribution; Table S2: GWAS analyses for each nutrient above and below DRI; Table S3: Interaction analyses for all nutrients and significant SNPs; Table S4: Single nucleotide polymorphism (SNP) showing significant interactions with nutrients associated with hypertension by gender.
