# Supplementary material for: Genome-Wide Approach of Gene–Nutrient Intake Interaction Study for Essential Hypertension in a Large Korean Cohort (KoGES)
Source: Nutrients. 2024 Nov 29;16(23):4147. doi: 10.3390/nu16234147 (PMC11644644; doi:10.3390/nu16234147)
Supplement: Supplementary file 1 [file nutrients-16-04147-s001.zip › Supplemental_Material.pdf]

Genome-Wide Approach of Gene-Nutrient Intake Interaction Study for Essential Hypertension in a Large Korean Cohort (KoGES)

Authors: Youhyun Song, MD, MMSc[1,2,*], Ja-Eun Choi, BS[3,*], Jae-Min Park, MD, MMSc[4], Yu-Jin Kwon, MD, PhD[5], Ji Won Lee, MD, PhD[6,7,#], and Kyung-Won Hong, PhD[3,#]

[*]Co-first authors

Affiliations:

[1] Department of Family Medicine, Gangnam Severance Hospital, Yonsei University College of Medicine, Seoul 06273, South Korea

[2] Healthcare Research Team, Health Promotion Center, Gangnam Severance Hospital, Yonsei University College of Medicine, Seoul 06273, South Korea

[3] Advanced Institute of Technology, Theragen Health Co. Ltd., Gyeonggi-do 13493, South Korea

[4] Department of Family Medicine, Uijeongbu Eulji Medical Center, Eulji University, Uijeongbu 11759, South Korea

[5] Department of Family Medicine, Yongin Severance Hospital, Yonsei University College of Medicine, Gyeonggi-do 16995, South Korea

[6] Department of Family Medicine, Severance Hospital, Yonsei University College of Medicine, Seoul 03722, South Korea

[7] Institute for Innovation in Digital Healthcare, Yonsei University, Seoul 06237, Republic of Korea

[#]Co-corresponding authors

Ji Won Lee, M.D., Ph.D.: Dept. of Family Medicine, Severance Hospital, Yonsei University College of Medicine, 50-1 Yonsei-ro, Seodaemun-gu, Seoul, 03722, South Korea. Tel: +82-2-2228-2330; Fax: +82-2-362-2473; E-mail: indi5645@yuhs.ac

Kyung-Won Hong, Ph D.: Advanced Institute of Technology, Healthcare R&D division, Theragen BioHealth Co. Ltd., A-10F, Samhwan HIPEX, 240, Pangyoyeok-ro, Bundang-gu, Seongnam-si, Gyeonggi-do, South Korea. Tel: +82-31-8017-9717; Fax: +82-31-288-1294; E-mail: kyungwon.hong@theragenhealth.com

SUPPLEMENTAL MATERIAL

Supplemental Table S1. Reference nutrient criteria and distribution.

➔ Refer to page 3 of this file.

Supplemental Table S2. GWAS analyses for each nutrient above and below DRI.

➔ Refer to Supplemental File S1

Supplemental Table S3. Interaction analyses for all nutrients and significant SNPs.

➔ Refer to Supplemental File S2

Supplemental Table S4. Single nucleotide polymorphism (SNP) showing significant interactions with nutrients associated with hypertension by gender.

➔ Refer to page 4 of this file.

Supplemental Table S1. Reference nutrient criteria and distribution.

Nutrients	Men			Women		
	Reference	Above Group, n (%)	Below Group n (%)	Reference	Above Group n (%)	Below Group n (%)
CHO, %	65	16,241 (83.5)	3210 (16.5)	65	26,304 (85.0)	4642 (15.0)
Protein, %	20	376 (1.9)	19,075 (98.1)	20	614 (2.0)	30,332 (98.0)
Fat, %	30	202 (1.0)	19,249 (99.0)	30	278 (0.9)	30,668 (99.1)
Ca, mg/day	737.5	1664 (8.6)	17,787 (91.4)	775	2756 (8.9)	28,190 (91.9)
P, mg/day	700	14,341 (73.7)	5110 (26.3)	700	20,992 (67.8)	9954 (32.2)
Iron, mg/day	9.5	9175 (47.2)	10,276 (52.8)	9.25	14,484 (46.8)	16,462 (53.2)
K, mg/day	3500	1772 (9.1)	17,679 (90.9)	3500	2882 (9.3)	28,064 (90.7)
Vitamin A, R.E	737.5	2884 (14.8)	16,567 (85.2)	612.5	6674 (21.6)	24,272 (78.4)
Na, mg/day	1350	16,112 (82.8)	3339 (17.2)	1350	24,104 (77.9)	6842 (22.1)
Vitamin B1, mg/day	1.15	6416 (33.0)	13,035 (67.0)	1	11,624 (37.6)	19,322 (62.4)
Vitamin B2, mg/day	1.425	1971 (10.1)	17,480 (89.9)	1.125	6784 (21.9)	24,162 (78.1)
Niacin, mg/day	14.75	8890 (45.7)	10,561 (54.3)	13.25	14,840 (48.0)	16,106 (52.0)
Vitamin C, mg/day	100	7601 (39.1)	11,850 (60.9)	100	14,327 (46.3)	16,619 (53.7)
Zinc, mg/day	9.5	5199 (26.7)	14,252 (73.3)	7.5	13,528 (43.7)	17,418 (56.3)
Vitamin B6, mg/day	1.5	9716 (50.0)	9735 (50.0)	1.4	16,318 (52.7)	14,628 (47.3)
Folate, mcg/day	400	1188 (6.1)	18,263 (93.9)	400	2056 (6.6)	28,890 (93.4)
Fiber, g/day	27.5	8 (0.04)	19,443 (99.96)	20	88 (0.3)	30,858 (99.7)
Vitamin E, mg/day	12	2700 (13.9)	16,751 (86.1)	12	4076 (13.2)	26,870 (86.8)
Cholesterol, mg/day	300	2361 (12.1)	17,090 (87.9)	300	3639 (11.8)	27,307 (88.2)

CHO, carbohydrate; K, potassium; P, phosphorus; R.E, retinol equivalents. Reference values of nutrients were based on following criteria by sex.
Acceptable macronutrient distribution range (AMDR); carbohydrate (%), protein (%), and fat (%). Recommended nutrient intake (RNI); Ca, P, Iron, K, Vitamin A, B1, B2, C, B6, Niacin, folate, zinc. Adequate intake (AI); potassium (K), sodium (Na), fiber, and vitamin E. Cholesterol was set as based on chronic disease endpoints.

Supplementary Table S4. Single nucleotide polymorphism (SNP) showing significant interactions with nutrients associated with hypertension by gender.

SNP	Chr: bp	Alleles		Positional Candidate Gene	Minor Allele Frequency				Coding Allele	Coding Allele Frequency	Gene and Nutrient Association results				Gene association result to Hypertension	
											Above Iron		Above Vit.B6			
		REF	ALT		Korean	EAS	EUR	AMR			OR (95% CI) (p-value)	Interaction p-value	OR (95% CI) (p-value)	Interaction p-value	OR (95% CI)	p-value
											Model 1					
rs13282715	8:4926756	T	A	CSMD1	0.065	0.062	0.202	0.117	A	0.040	0.72 (0.64-0.81) **(4.86×10^{-8})**	1.34×10^{-3}	0.73 (0.66-0.82) **(4.12×10^{-8})**	7.18×10^{-4}	0.88 (0.82-0.95)	9.21×10^{-4}
											Model 2					
											0.74 (0.64-0.87) (1.49×10^{-4})	1.26×10^{-1}	0.74 (0.64-0.86) (6.22×10^{-5})	1.38×10^{-1}	0.89 (0.81-0.98)	1.83×10^{-2}
											Model 3					
											Male					
											0.72 (0.62-0.85) (1.30×10^{-4})	4.26×10^{-1}	0.73 (0.63-0.86) (1.30×10^{-4})	4.44×10^{-1}	0.84 (0.76-0.93)	1.14×10^{-3}
											Female					
											0.72 (0.61-0.85) (1.21×10^{-4})	1.10×10^{-2}	0.73 (0.63-0.85) (8.17×10^{-5})	1.06×10^{-2}	0.93 (0.84-1.03)	1.45×10^{-1}

SNP, single nucleotide polymorphism; Chr, chromosome; BP, base pair; EAS, East Asian; EUR, European; AMR, American; OR, odds ratio; CI, confidence interval. In Model 1, P-values were calculated using the logistic regression analysis adjusting for age, sex, exercise, smoking, alcohol intake (g/day), total calorie consumption, PC1, and PC2. Interaction p-values were calculated through the interaction term of the general linear regression model. In Model 2, Type 2 diabetes mellitus and dyslipidemia were included as additional covariates in the calculations for P-values and interaction P-values, based on Model 1. In Model 3, results were analyzed separately for males and females under the same conditions as Model 1.
